# Aging population challenges: enhancing healthy aging practices through virtual coaching systems

**DOI:** 10.1186/s12913-025-13497-9

**Published:** 2025-10-01

**Authors:** Marta Pinzone, Francesco Paolo Appio, Luca Gastaldi, Emanuele Lettieri

**Affiliations:** 1 Department of Management, Economics and Industrial Engineering, Politecnico di Milano, Piazza Leonardo da Vinci 32, Milan, 20133 Italy; 2 Paris School of Business, Paris, France

**Keywords:** Digital technologies, Virtual coaching systems, Technology acceptance model, Healthy aging, Structural equation modelling

## Abstract

**Background:**

As the global population ages, society faces complex challenges in healthcare provision and medical service delivery. This study focusses on the potential of supportive digital technologies, with a particular focus on Virtual Coaching Systems (VCSs), to offer innovative solutions for improving healthy aging practices. Our research extends the existing Technology Acceptance Model (TAM) by incorporating three novel external determinants: subjective norms, health literacy, and information technology literacy. These additional factors provide a more nuanced understanding of the drivers behind technology acceptance among older adults, highlighting the importance of addressing the unique needs and preferences of this population segment.

**Methods:**

Drawing on empirical evidence from a sample of 436 Italian individuals aged 60 and above, we develop a comprehensive framework and test a structural equation model that unveils the crucial role of exploiting diverse knowledge sources through VCSs in promoting healthy aging, ultimately contributing to the sustainability of national healthcare systems.

**Results:**

In line with the TAM, perceived usefulness and perceived ease of use have a positive and significant impact on the elderly people’s intention to use a VCS. Subjective norms are not found to have a significant direct impact on the intention to use a VCS, while they have an indirect influence through perceived ease of use and perceived usefulness. Health literacy is found to have a significant but negative impact on the perceived usefulness of a VCS. On the other hand, it positively and significantly affects perceived ease of use. Finally, the results of the analysis provide support for the positive relationship between information technology literacy and perceived ease of use.

**Conclusions:**

Our findings offer valuable insights for healthcare researchers, policymakers, professionals, and digital solution providers in developing targeted strategies and interventions to support the health and well-being of the aging population. This study emphasizes the need for a collaborative approach among stakeholders to harness the potential of VCSs in addressing the multifaceted challenges arising from population aging, ultimately enhancing the quality of life for older individuals and promoting the sustainability of healthcare systems worldwide.

## Introduction

As populations age, promoting healthy aging and enhancing healthcare delivery for older adults has become a global priority [1–5]. Digital technologies, particularly Virtual Coaching Systems (VCSs), offer promising solutions to these challenges by integrating and managing professional knowledge [6–10] to support healthy aging in a cost-effective way [11–15]. Powered by Artificial Intelligence (AI) and diverse data sources, VCSs deliver personalized support through real-time monitoring, feedback, and tailored recommendations [13, 16–19]. Key characteristics of VCSs include [11, 13, 16, 20, 21]: social ability to maintain user relationships, credibility and trustworthiness, context awareness for goal alignment, personalized advice through continuous learning, data collection and analysis (e.g.,., sensors, mood, sleep), proactivity in user engagement, use of behavior change models, support for future-oriented planning.

VCSs are gaining traction in both clinical and everyday settings [11, 14, 17, 22]. These systems have been applied to a variety of healthy ageing domains, including promoting and improving the levels of physical activity (e.g., [23–25]), nutrition (e.g., [20, 21, 26, 27]), physical activity motivation for the elderly individuals [28, 29], social interactions or cognitive maintenance over time [30].They have shown effectiveness across age groups [17] and offer scalable, cost-effective solutions for promoting healthy aging [31, 32].

Despite the growing reliance on digital technologies among older adults, the adoption of VCSs among those aged 60 and above remains low [33, 34]. Understanding the intentional use of VCSs is essential to uncover how these systems can effectively support healthy aging behaviors. Unlike traditional e-health systems, which primarily focus on medical care and health management, VCSs are distinguished by their emphasis on overall well-being, lifestyle improvement, and personalized, continuous support. Therefore, this research aims to shed light on the factors influencing individuals aged 60 + to adopt VCSs. Its central research question is:

***Which are the determinants of adopting VCSs to enhance healthy aging practices?***

To answer this research question, we developed and tested a theoretical model grounded in the original Technology Acceptance Model (TAM) formulation by Davis [35]. TAM is a widely recognized framework in healthcare research for examining the factors that affect the adoption of health technologies (e.g., [36–38]), and has been extensively applied in gerontology [39, 40]. Although TAM has undergone several modifications that have resulted in the development of different models, such as the TAM2 and the UTAUT [41], the original formulation by Davis [35] remains the most widely used by scholars across disciplines due to its proven robustness, predictive power, and cost-effectiveness [42–45].

Consistent with TAM, we considered behavioral intention to use as the dependent variable in our model, as it is a strong predictor of actual usage (e.g., [44]). Moreover, according to TAM, two primary antecedents influence an individual’s behavioral intention to use technology for a specific goal: perceived usefulness and perceived ease of use [35]. Perceived usefulness represents the extent to which an individual believes using a system will enhance their performance, while perceived ease of use signifies the degree to which a person believes using a system will be easy, allowing to avoid difficulties and complications.

In the context of VCSs, both are expected to positively influence intention. This is consistent with the cognitive rationale that individuals prefer technologies that address their needs efficiently while minimizing the learning curve associated with their usage. Prior studies confirm these relationships in the adoption of wellness devices and health information systems [46–48]. Therefore, it is reasonable to advance the following two hypotheses:

### H_1_:

Perceived ease of use has a positive influence on the intention to use a VCS for healthy ageing.

### H_2_:

Perceived usefulness has a positive influence on the intention to use a VCS for healthy ageing.

Furthermore, TAM proposes that perceived ease of use has a positive effect on perceived usefulness. This means that if an individual perceives a technology as easy to use, they are more likely to perceive it as useful in achieving their goals. This relationship has been widely confirmed by previous studies in healthcare (e.g., [39, 49, 50]). Therefore, based on the existing literature and the foundational premises of TAM, it is reasonable to advance the following hypothesis:

### H3:

Perceived ease of use has a positive influence on the perceived usefulness of a VCS for healthy ageing.

In this study we expanded TAM’s original formulation by examining the role of three additional determinants, which have been found significant in the literature for health-related behaviors and are central to improvement strategies implemented by healthcare systems worldwide: (1) subjective norms, (2) health literacy, and (3) information technology literacy.

Subjective norms refer to the social influence exerted by individuals perceived as important by a person. Research shows that these norms significantly impact the intention to adopt new technologies especially when influenced by trusted individuals such as family or healthcare providers [51]. In the context of VCSs for healthy aging, this influence is particularly relevant, as older adults may lack digital experience and rely on opinions of their family members and doctors (e.g., [52]). However, this trend is evolving. According to the World Economic Forum, 70% of seniors are now online, reflecting a growing engagement with digital technologies. The opinions of others can positively influence an individual’s intention to use VCSs by affecting their perceptions of the technology’s ease of use and usefulness (e.g., [53, 54]). These opinions can be useful in developing a deeper understanding of the technology’s nature, features, and functionalities (e.g., [55, 56]).

Based on this evidence, it is reasonable to hypothesize that subjective norms will have a positive influence on an individual’s intention to use a VCS for healthy ageing. This influence may be partially mediated by perceived usefulness and perceived ease of use. Therefore, it is reasonable to advance the following hypothesis:

### H_4_:

Subjective norms have a positive influence on the intention to use a VCS for healthy ageing with a partial mediation of (a) perceived usefulness and (b) perceived ease of use.

Health literacy is related to the capability of an individual to obtain, comprehend, apply, and communicate health information to make informed decisions [57]. Individuals who possess those skills will require little effort to use technological devices for their healthy aging needs [54, 58, 59]. According to Mackert et al. [57], American people with a higher health literacy perceived health information technology to be more useful and easier to use. Similarly, the perceived ease of using online health apps was found to be related to the ability to gather and interpret health information in Singapore and Taiwan [52, 60].

In the context of VCSs, recent research (e.g., [61, 62]. , suggest individuals with high levels of health literacy are likely to perceive a VCS as easier to use and more useful in helping them achieve their healthy ageing goals. Therefore, based on the existing literature and recent research on the topic, we make the following hypothesis:

### H_5_:

When it comes to adopt a VCS for healthy ageing, health literacy has a positive influence on both the (a) perceived usefulness and (b) perceived ease of use.

Information technology literacy refers to an individual’s ability to use digital devices [63]. In the context of VCSs for healthy ageing, it is reasonable to hypothesize that information technology literacy will positively affect an individual’s intention to use such a system through the mediation of perceived ease of use (e.g., [62]). This, in turn, may increase their intention to use the system. Thus, we can advance this last hypothesis:

### H_6_:

Information technology literacy positively affects intention to use a VCS for healthy ageing through the mediation of perceived ease of use.

**Fig. 1:**
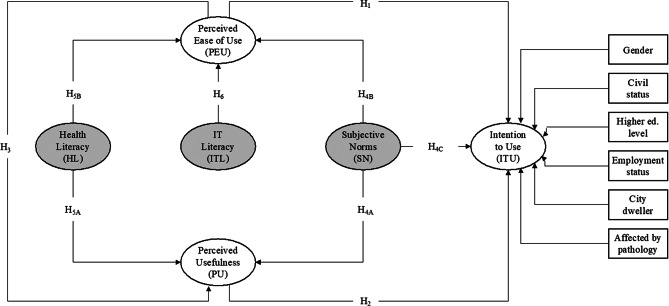
Theoretical model and hypotheses

Overall, Fig. 1 presents our theoretical framework and its main hypotheses. In addition, it shows the control variables included in the study: gender (male, female), civil status (married, not married), higher education level (has, does not have), employment status (employed, unemployed), whether the elderly individual lives in a city, and whether the elderly individual is affected by a pathology.

## Methodology

### Sample and data collection

To rigorously test our hypotheses, within the framework of NESTORE H2020 project^1^, data were collected from individuals aged 60 and above in Italy. We employed a robust sampling strategy to collect data from individuals aged 60 and above in Italy. From June to August 2018, we administered an online, anonymous, voluntary survey written in Italian to ensure accessibility and ease of participation for elderly respondents. Considering an effect size of 0,2, with a significance level (α) of 0,05 and a desired statistical power of 0,80, the minimum sample size was established at 403 responses [73]. The survey was created using Qualtrics software. The online survey was designed using simple language and an accessible format, featuring large fonts, intuitive navigation, and clear instructions. The questionnaire was divided into two main sections: the first focused on gathering general information about the respondent, while the second aimed to collect data for measuring the specific constructs of our model. To reduce respondent burden, the items were distributed across six pages. A pilot test was conducted to identify and resolve any confusing questions or technical issues. The survey was distributed through multiple channels, including:

- The website and newsletter of Grey Panthers, an Italian online platform on healthy ageing targeting seniors since 2008. The survey was published on their website and shared via their newsletter.
- Facebook social media platform, which was chosen because it is the most popular one among seniors.
- Word of mouth to maximize the reach and diversity of our sample (including variation in gender, education levels, geographic location, etc.).

All data collection and processing procedures were conducted in strict compliance with the European General Data Protection Regulation to ensure the protection of personal data. IP addresses were used to avoid potential duplicate entries. The survey took an average of 15 min to complete. Participants were allowed to review and modify their responses before final submission. All responses were automatically saved in the Qualtrics database.

### Measures

All constructs were measured using previously published scales (Table 1). Unless otherwise specified, responses were given on a five-point Likert scale ranging from ‘strongly disagree’ (1) to ‘strongly agree’ (5). Table 1 shows the various constructs, items, and Cronbach’s alpha values.

**Table 1:** Constructs and relative items

Constructs^A^	Items	Cronbach’s α
Intention to use (ITU) [78]	• ITU_1_: I would consider the use of solutions and new technologies that would guide me in enhancing my lifestyle and daily activities • ITU_2_: I would consider the use of a mobile application that guides me towards the maintenance of my physical wellbeing • ITU_3_: I would consider the use of a mobile application that would guide me towards the maintenance of my cognitive wellbeing • ITU_4_: I would consider the use of a mobile application that would guide me towards the maintenance of my social wellbeing and of my relations • ITU_5_: I would consider the use of a mobile application that would guide me towards the maintenance of my cognitive abilities • ITU_6_: I would consider the use of a mobile application that would guide me towards the control and management of my eating habits	0.9126
Perceived Ease of Use (PEU) [35]	• PEU_1_: Using applications for the control of the lifestyle would be easy for me • PEU_2_: I would be able to use solutions and new technologies to control of my lifestyle in daily activities • PEU_3_: I would have the right competencies required to use solutions and new technologies for the control of my lifestyle in my daily activities • PEU_4_: I would have the resources required to use solutions and new technologies for the control of my lifestyle in my daily activities	0.8273
Perceived Usefulness (PU) [35]	• PU_1_: Using a digital application for the control of health would bring benefits to my wellbeing • PU_2_: Using a mobile application for the management of the health status would make me more active and conscious in taking decisions concerning health	0.8550
Subjective Norm (SN) [51]	• SN_1_: My relatives think that it would be useful for my health to use a mobile application that helps me to control my lifestyle • SN_2_: My doctor thinks that it would be useful for my health to use a mobile application that helps me to control my lifestyle • SN_3_: My friends think that it would be useful for my health to use a mobile application that helps me to control my lifestyle	0.8961
Health Literacy (HL) [79]	• HL_1_: I find it easy to find out how to maintain my physical wellbeing • HL_2_: I find it easy to find out how to maintain a balanced diet • HL_3_: I find it easy to find information concerning the activities that can have a positive impact on my wellbeing (e.g.,. meditation, gymnastic courses, walking) • HL_4_: I find it easy to understand the daily behaviour (eating habits or physical exercise) that are related to my health	0.8087
IT Literacy (ITL) [80]	Two items obtained by counting the number of Apps used by the respondent • ITL_1_: Which of the following apps do you use? Information, Fitness, Nutrition, Brain Game, Web Surfing • ITL_2_: Which of the following social network do you use? Facebook, Whatsapp, Instagram, Twitter, Snapchat	0.7349
6 binary control variables	Gender (male, female); Civil Status (married, not married); Higher education level (has, has not); Employment status (employed, unemployed); City dweller (yes, no); Affected by pathology (yes, no)	

^A^ The theoretical sources of the various constructs are represented in parentheses

^B^ For each item it was asked how the respondedt agreed with the relative sentence on a scale from 1 to 5

(1 = strongly disagree; 2 = agree; 3 = neither agree nor disagree; 4 = agree; 5 = strongly agree)

Our model includes several control variables, including gender (male, female), civil status (married, not married), higher education level (has, does not have), employment status (employed, unemployed), whether or not the elderly individual lives in a city, and whether the elderly individual is affected by a pathology.

### Data analysis

Our model was tested through structural equation modelling using the statistical software STATA v14 [64–66]. The validity of constructs and the significance of hypothesized relationships among the latent variables were respectively verified through the output of the measurement and the structural model. As a final step, the goodness of fit of the proposed model was assessed considering both absolute and relative fit indexes. Four indexes were considered as recommended by the academic literature, i.e., the Root Mean Square Error of Approximation (RMSEA), the Standardized Root Mean Residual (SRMR), the Comparative Fit Index (CFI), the Tucker-Lewis Index (TLI). As reported in Table 2, all indexes’ values are in line with common values accepted in the literature (e.g., [67]).

**Table 2:** Goodness of fit indexes

Index	Value	Suggested Threshold
RMSEA	0.06	< 0.07
SRMR	0.05	< 0.08
CFI	0.91	> 0.90
TLI	0.91	> 0.90

## Results

The survey yielded a total of 560 responses. A rigorous quality check to remove inconsistent or incomplete responses was performed. To have a satisfactory quality of the input data, the answers containing inconsistent or missing values for more than 40% of the attributes were discarded. Therefore, our final sample comprised 436 high-quality responses, exceeding the minimum recommended sample size of 403 observations suggested by [73] to detect medium/small effect sizes, with a significance level of α = 0,05 and a desired power of 0,80. The final sample was characterized by a diverse range of demographic attributes: 37% of respondents were male, 46% had completed post-secondary education, 45% were still employed, and 68% were married or cohabiting.

Table 3 displays the Average Variance Extracted (AVE) and Composite Reliability (CR) for each construct in the structural equation model. The values are higher than commonly accepted thresholds (equal to 0.50 and 0.70, respectively) indicating good validity for all constructs [67–69].

**Table 3:** Confirmatory factor analysis (CFA)

Construct	Id	Factor Loading	AVE	CR
Intention To Use (ITU)	ITU_1_	0.7464	0.6420	0.9146
	ITU_2_	0.8546		
	ITU_3_	0.8541		
	ITU_4_	0.7243		
	ITU_5_	0.8275		
	ITU_6_	0.7908		
Perceived Usefulness (PU)	PU_1_	0.8892	0.7105	0.8303
	PU_2_	0.7939		
Perceived Ease of Use (PEU)	PEU_1_	0.7850	0.5973	0.8549
	PEU_2_	0.8672		
	PEU_3_	0.7374		
	PEU_4_	0.6909		
Subjective Norm (SN)	SN_1_	0.8377	0.7520	0.8746
	SN_2_	0.8372		
	SN_3_	0.9238		
Health Literacy (HL)	HL_1_	0.7336	0.5227	0.8136
	HL_2_	0.6497		
	HL_3_	0.7452		
	HL_4_	0.7584		
IT Literacy (ITL)	ITL_1_	0.7619	0.6083	0.7564
	ITL_2_	0.7976		

**Fig. 2:**
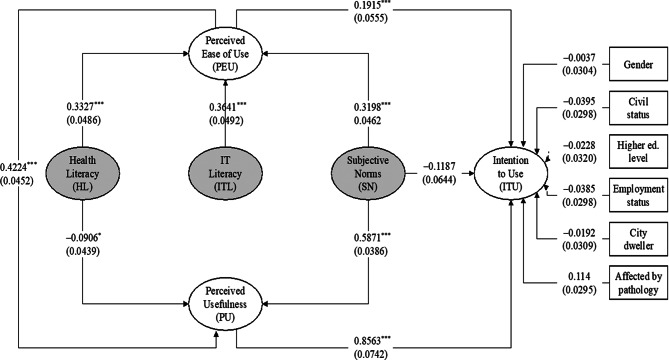
Structural equation model. Notes: Standardized coefficients are reported, with standard errors in the parentheses. * *p* < 0.1; ** *p* < 0.05; *** *p* < 0.01

Figure 2 shows the structural model with tested relationships and control variables. Standardized path coefficients are reported, with standard errors in the parentheses.

None of the control variables appear to influence the intention to use a VCS. The path coefficients for our TAM-related hypotheses, on the other hand, show that both perceived usefulness (β = 0.86; *p* ≤ 0.001) and perceived ease of use (β = 0.19; *p* ≤ 0.001) have a positive and significant impact on the respondent’s intention to use a VCS. As a result, H_1_ and H_2_ are supported. Furthermore, the results show that perceived ease of use has a positive and significant effect on perceived usefulness (β = 0.42; *p* ≤ 0.001), indicating that H_3_ is also supported.

Concerning subjective norms, the result shows that H_4_ is partially supported. Subjective norms are not found to have a significant direct impact on the intention to use a VCS (β = 0.12; *p* > 0.1), while they have an indirect influence through perceived ease of use (β = 0.32; *p* ≤ 0.001) and perceived usefulness (β = 0.59; *p* ≤ 0.001).

Similarly, the data partially support H_5_. On one hand, and contrary to our expectations, health literacy is found to have a significant but negative impact on the perceived usefulness of a VCS (β = 0.09; *p* ≤ 0.05). On the other hand, it positively and significantly affects perceived ease of use (β = 0.33; *p* ≤ 0.001).

Finally, the results of the analysis provide support for the positive relationship between information technology literacy and perceived ease of use (β = 0.36; *p* ≤ 0.001). Therefore, H_6_ is supported.

## Discussion and conclusions

### Theoretical contributions

The decision to accept and use VCSs by older people lags far behind the speed of creation of these devices [11, 12, 70]. Despite it is acknowledged that older adults’ adoption of VCSs and healthy ageing behaviors is a rather complex issue with multiple individual, technical, social aspects to consider, research on this topic has received limited attention thus far (e.g., [62]). In contrast, most existing literature and research endeavours have primarily centred around the design, development and testing of novel VCSs (e.g., [61, 71, 72]). Such research gap corroborates the need to focus on identifying the factors that explain the process of acceptance and adoption of VCSs by older adults [40].

Our findings provide substantial support for the basic linkages within the TAM (29). Specifically, our research confirms that higher perceived ease of use and perceived usefulness of a VCS lead to increased intentions to use the technology. These findings align with previous research applying TAM to healthcare contexts (e.g., [36–38, 42]).

An original contribution we discovered is the non-significant direct relationship between subjective norms and the intention to use a VCS. Contrary to our hypothesis, this result implies that the degree to which important people in the lives of elderly individuals (such as friends, family members, and doctors) do not directly influence VCS adoption. This finding is consistent with the findings of Lee and Lee [32] in South Korea, who discovered that interpersonal influences have an indirect impact on the intention to use a wearable fitness tracker. Similarly, we discovered that perceived usefulness and perceived ease of use completely mediate the relationship between subjective norms and the intention to use a VCS. Given the target population and the voluntariness of using a VCS, we can assume that the positive (or negative) opinions of friends, family members, and doctors influence the intention to use only when they are fully internalized in the belief structure of elderly individuals [51]. Furthermore, the availability of assistance and guidance from these important individuals may contribute to the perception that using the technology is manageable and straightforward. Our study extends this understanding by exploring the specific nature of these mediating relationships in the context of VCS usage among the elderly, thereby contributing to a deeper understanding of the social dynamics at play in technology adoption in this demographic.

Contrary to expectations, we uncovered a novel finding that health literacy has a negative impact on the perceived usefulness of a VCS. A potential interpretation of this finding is that, when an elderly individual already possesses the ability to effectively access and comprehend information related to their personal well-being, they may perceive the use of an additional digital tool, such as a VCS, as unnecessary and offering limited value for maintaining their well-being. These findings align with Neter and Brainin [73], who reported that individuals with higher health literacy tended perceiving less usefulness of digital health tools, as they were already capable of managing their health independently. This insight challenges conventional wisdom in digital health, suggesting a more nuanced understanding of the relationship between personal capabilities and technology adoption, particularly in the context of elderly users.

These findings provide preliminary evidence for a boundary condition that modifies (albeit slightly) the provisions of TAM. In other words, we can argue that TAM is not a one-size-fits-all model for all technological solutions. Rather, specific technological solutions – such as VCSs – appear to act as boundary conditions to its applicability. Kim and Park [74] also argued for the need to consider context-specific factors when applying TAM to different technologies, as the model’s assumptions may not hold true in all cases. This study contributes to this debate by offering empirical evidence of the variability in the applicability of TAM, especially in the context of supportive technologies for the elderly.

The domain of supportive digital technologies, of which VCSs are only one example, typically have an inherent high social value and may necessitate more in-depth future investigation into the nature of TAM. For instance, Griebel et al. [75] emphasized the importance of understanding users’ perspectives and the influence of external factors when examining the adoption of digital health technologies. Our research adds to this body of work by specifically focusing on the elderly demographic, underlining the unique factors influencing their adoption of digital technologies.

Moreover, our findings confirm that a higher level of health literacy leads elderly people to perceive the VCS as easier to use. This finding is consistent with previous research in the field of e-health, such as the study by Cho et al. [53] in the US, which demonstrated that higher health literacy was associated with greater perceived ease of use of health-related technologies. In summary, our research contributes to the growing body of literature on the adoption of supportive digital technologies, highlighting the importance of considering health literacy and other context-specific factors when applying TAM to these technologies. In extending these findings, our study not only corroborates existing literature but also offers a novel perspective on the complex interplay of health literacy and technology use, particularly in the aging population.

Lastly, our results provide strong support for the positive relationship between intention to use and perceived ease of use of a VCS. This finding supports theoretical arguments that a high level of familiarity with technological devices increases the ease of use of personal health and lifestyle digital systems (e.g., [32, 76]). Our study adds to this understanding by specifically examining this relationship within the context of VCSs, thereby reinforcing the relevance of ease of use as a critical factor in the adoption of new technologies by older adults.

Overall, our research contributes to the growing body of literature on the adoption of supportive digital technologies, highlighting the importance of considering health literacy and other context-specific factors when applying TAM to these technologies. Our work, therefore, represents a significant step forward in understanding the technology adoption process among the elderly, thereby adding a new dimension to the ongoing discourse in this field.

### Practical contributions

Our results highlight important contribution for health policymakers, healthcare professionals, and providers of digital solutions for the healthy aging of the elderly. The first is to design and sponsor a solution that appears simple to use and in which the VCS is perceived as simple to follow. This recommendation is directly informed by our empirical evidence. Therefore, emphasizing a user-friendly interface with minimal steps and data requirements is not just about aesthetics; it is about aligning with the psychological predispositions of the target user group.

This goal can be met by demonstrating that only a few steps are required to interact with the system and communicating that only a small amount of data will be required to begin the coaching program. A graphical interface that conveys the idea of simplicity may increase this perception even more. Simplifying the interaction process enhances perceived ease of use, which in turn, significantly increases the likelihood of VCS adoption among older adults. Hence, designing with simplicity in mind is not merely a design principle but a strategic approach grounded in our findings.

Second, health professionals and policymakers should emphasize the actual utility of these VCSs to users to increase their adoption. They should take steps to raise awareness of the actual benefits of virtual coaching among the elderly, such as by sponsoring successful cases of application. This approach is validated by our discovery that perceived usefulness is a critical determinant of technology acceptance. Showcasing real-life success stories can effectively communicate the tangible benefits of VCS, addressing the identified gap between health literacy and perceived usefulness.

Similarly, in the case of health literacy and its negative impact on the perceived usefulness, system developers and healthcare professionals must provide clear evidence of the effective benefits and value added driven by such systems’ ability to act on an individual’s behavior. The knowledge used by the VCS must come from professional sources and go beyond what citizens learn through their own research. Our findings indicate that overcoming scepticism among highly health-literate elderly requires demonstrating the unique advantages of VCS that cannot be replicated through conventional health management practices.

Beyond generic recommendations, Artificial Intelligence (AI) must be sophisticated enough to personalize feedback and advice to a single user (e.g., 10,000 steps a day). AI can aid in the popularization of VCSs in a variety of ways, including engaging elderly individuals through micro-learning sessions, gamification, and the development of motivational and inspirational apps. The call for AI sophistication is grounded in our study’s insights into the importance of personalized and relevant interactions to foster VCS adoption. Specifically, our research suggests that personalization through AI significantly enhances perceived ease of use and usefulness, thereby promoting deeper engagement and sustained use among elderly individuals. AI-enabled coaches will grow in popularity, reaching a level of sophistication where elderly individuals can receive real coaching conversations on specific topics. Based on user-based AI measures, VCSs and comfortable human–computer interfaces have the potential to promote active information processing and adoption in terms of motivation and behavior changes [77]. Our study supports the notion that interactive and responsive VCSs, enabled by advanced AI, could align closely with the preferences and needs of the elderly demographic, thus enhancing the practical utility of these systems.

Finally, policymakers should encourage the development of educational programs to better equip elderly people with the digital skills they need to use VCSs and avoid exacerbating the “digital divides” that unethically exclude those with low information technology literacy (e.g., [10, 74]). Tailored educational programs that enhance digital literacy among the elderly are crucial for ensuring equitable access and maximizing the benefits of VCS for all users, thereby addressing a key barrier identified in our research.

### Limitations and future research

First, because we used a cross-sectional design, we cannot draw definitive conclusions about causation. Although previous contributions aided in the development of our hypotheses, longitudinal research would be beneficial in determining causality.

Second, data were gathered through an online survey. Even if the issue does not appear to be as pressing as it once was – as the World Economic Forum recently demonstrated^2^ – it still implies that elderly people with limited internet access or low digital skills may have been underrepresented in the final sample, which may not be perfectly representative of the entire population. Furthermore, as our data were collected in a single country (i.e., Italy), the generalizability of the findings should be approached with caution. Nonetheless, the constructs derived from the extended TAM have been studied and validated across diverse international contexts and the power analysis confirms that our sample size (*N* = 436) is sufficient to detect medium/small effect sizes, with a significance level of α = 0,05 and a desired power of 0,80. This suggests that the model’s applicability may extend beyond national boundaries, particularly to settings with similar socio-demographic and healthcare characteristics. Future research conducted in different countries is encouraged to further validate and refine the generalizability of these findings. Finally, because of the unexpected negative effect of health literacy on VCS intention to use, and because our construct focused on the wellbeing information domain, it may be worthwhile to investigate the role of health literacy further, taking other dimensions into account.

Despite these limitations, our results not only support existing empirical evidence but also shed new light on the role of determinants and the inner mechanisms that shape elderly individuals’ intention to use a VCS for healthy ageing practice. In doing so, it contributes to a theoretical and practical understanding of users’ discretionary acceptance of supportive digital technologies as a means of achieving healthy-ageing-related goals.

## Data Availability

The datasets used and/or analysed during the current study are available from the corresponding author on reasonable request.

## Footnotes

1
https://nestore-coach.eu/home.

2
https://www.weforum.org/agenda/2019/07/no-longer-just-for-the-young-70-of-seniors-are-now-online.

## Abbreviations

AI: Artificial Intelligence
AVE: Average Variance Extracted
CFI: Comparative Fit Index
CR: Composite Reliability
HL: Health Literacy
ITL: IT Literacy
ITU: Intention To Use
PEU: Perceived Ease of Use
PU: Perceived Usefulness
RMSEA: Root Mean Square Error of Approximation
SN: Subjective Norm
SRMR: Standardized Root Mean Residual
TAM: Technology Acceptance Model
TLI: Tucker-Lewis Index
UTAUT: Unified Theory of Acceptance and Use of Technology
VCS: Virtual Coaching System
